# Self-Medication with Antibiotics, Attitude and Knowledge of Antibiotic Resistance among Community Residents and Undergraduate Students in Northwest Nigeria

**DOI:** 10.3390/diseases6020032

**Published:** 2018-04-27

**Authors:** Olumide Ajibola, Olusola Akintoye Omisakin, Anthonius Anayochukwu Eze, Semeeh Akinwale Omoleke

**Affiliations:** 1 Department of Microbiology, Faculty of Science, Federal University, Birnin Kebbi, Kalgo Road P.M.B. 1157, Birnin Kebbi 860222, Kebbi State, Nigeria; olumide.ajibola@fubk.edu.ng; 2 Department of Demography and Social Statistics, Faculty of Art, Social and Management Sciences, Federal University Birnin Kebbi, Kalgo Road P.M.B. 1157, Birnin Kebbi 860222, Kebbi State, Nigeria; omisakinolusola@yahoo.com; 3 Department of Medical Biochemistry, University of Nigeria, Enugu Campus, Enugu 400241, Nigeria; 4 Immunization, Vaccines and Emergencies, World Health Organization, Kebbi State Field Office, Birnin Kebbi 860222, Nigeria

**Keywords:** self-medication, antibiotic resistance, community, undergraduates

## Abstract

This study set out to evaluate self-medicated antibiotics and knowledge of antibiotic resistance among undergraduate students and community members in northern Nigeria. Antibiotic consumption pattern, source of prescription, illnesses commonly treated, attitude towards antibiotics, and knowledge of antibiotic resistance were explored using a structured questionnaire. Responses were analyzed and summarized using descriptive statistics. Of the 1230 respondents from undergraduate students and community members, prescription of antibiotics by a physician was 33% and 57%, respectively, amongst undergraduate students and community members. We tested the respondents’ knowledge of antibiotic resistance (ABR) and found that undergraduate students displayed less knowledge that self-medication could lead to ABR (32.6% and 42.2% respectively). Self-medication with antibiotics is highly prevalent in Northwest Nigeria, with most medicines being purchased from un-licensed stores without prescription from a physician. We also observed a significant gap in respondents’ knowledge of ABR. There is an urgent need for public health authorities in Nigeria to enforce existing laws on antibiotics sales and enlighten the people on the dangers of ABR.

## 1. Introduction

Increase in antibiotic resistance (ABR) worldwide, specifically in developing countries, necessitates the need to pay attention to self-medicated antibiotics, and knowledge and awareness of ABR [1,2]. In May 2015, the World Health Assembly reached an agreement to tackle the menace of ABR globally, and the first objective was to increase ABR awareness and understanding [3]. Antibiotics are medicines formulated for treatment or prevention of bacterial infections, administered to patients based on the prescription of a certified health care professional. In developing countries, antibiotics can be readily purchased without any control; such countries usually experience more cases of ABR, in contrast to what occurs in western nations where tight regulations of antibiotic use are in place [4]. Self-medication can be defined as the use of drugs to treat self-diagnosed illnesses or symptoms or the continued use of prescribed drugs for chronic or recurrent diseases or symptoms [5]. Self-medication practices include acquiring medicines without prescription, reusing old prescriptions to purchase medicines, or the sharing of medicines with relatives, friends, or immediate family members [6]. In developing countries, especially in remote areas, self-medication practices are the norm. Self-medication practices have a major pitfall, which is that users do not follow a prescribed course of drug dosage and usually stop the drug regimen once they are relieved of symptoms of illness. Studies have shown that self-medication is a common practice among healthcare workers, including doctors [7,8]. In Nigeria, there is poor regulatory control of antibiotic sales, and purchases can be carried out without the prescription of a clinician. In addition, there is a preponderance of counterfeit drugs in the market which makes it difficult to achieve effective treatment of self-diagnosed illnesses.

The spread of ABR is known to be associated with inadequate dosing, incomplete courses, and counterfeit drugs which increases the actual cost of treating illnesses and social burden. It is important that control on sale and self-medication with antibiotics should be tightened to reduce the problem of ABR. In Nigeria the prevalence of self-medication is very high, and there are reports of the use of antibiotics for minor ailments, cold, and menstrual pain [7,9,10,11,12]. With increased access to the internet and regular adverts of pharmaceutical products on television in Nigeria, young people are highly prone to self-medication with antibiotics. In sub Saharan Africa, ABR to common clinical isolates is on the increase, including in Nigeria [13,14]. Estimates suggests that 700,000 lives are lost annually to ABR, and it is projected to lead to mortality of at least 10 million lives by 2050 [15]. ABR leads to increased health care costs, longer hospital stays, and limited drug options for treatment, most especially in developing countries. Globally, the WHO is working towards raising awareness of ABR and increasing antibiotic stewardship.

To this end, the aim of this study was to assess the prevalence and pattern of self-medication with antibiotics amongst undergraduate students enrolled in a Federal university, and community members resident in Kebbi State, Northwest Nigeria. In addition, we investigated the respondents’ knowledge of ABR and attitude towards antibiotics use. This research is motivated by the alarming spread of ABR in developing countries, the paucity of data in Northwest Nigeria on self-medication with antibiotics, and the alarming rate of drug hawkers and patent medicine stores that sell antibiotics and prescription-only medicines that are unauthorized to do so. We hope that the findings here will provide public health practitioners with data to design a line of action to ameliorate the alarming trends of self-medication with antibiotics and ABR in our setting.

## 2. Methods

### 2.1. Study Site

This study was carried out in Kebbi, a state in Northwest, Nigeria. The state’s demographic features include mainly individuals from the Hausa ethnic group and Islamic religion, comprising 21 local government areas (LGA), and four emirates namely Gwandu, Argungu, Yauri and Zuru. Kebbi has an estimated population of 4,531,129 based on 2006 projected census figures on a total area of 36,800 km^2^. Nigeria is located in West Africa and made up of approximately 180 million people, comprising three major ethnic groups, Yoruba, Igbo, and Hausa. The people of Kebbi belong mainly to the Hausa ethnic group, and the three ethnic groups have similar life style patterns and religious beliefs consistent with most parts of Northern Nigeria.

### 2.2. Study Design and Data Collection

This cross-sectional study was carried out between July–August 2017 with respondents drawn from the halls of residence of the Federal University Birnin Kebbi, Kebbi (public university) with a student population of 3500 students and residents of the 21 LGAs in the state. The Federal University is located in Kalgo LGA and was established in 2013 with three faculties and 13 departmental programmes currently. For community respondents, Kebbi state government health care facilitators at each LGA in Kebbi were informed of the study, and trained and assisted with collecting data from each LGA they represent, based on convenience and proximity to place of work. Prior to data collection, the structured questionnaire had been pre-tested before the actual date of questionnaire administration, and contained information such as socio-demographic characteristics of the study participants, illnesses for which self-medication was sought, sources of advice, frequency of antibiotic use, reasons for antibiotic use, types of antibiotics used, knowledge of ABR, and liquids used to take antibiotics. The question on liquids used to take antibiotics was asked because it has been documented that juice and other liquids apart from water could potentially interact with drugs and limit their absorption in the body [16,17,18]. Questionnaires administered to both community residents and undergraduate students was presented in the English language, and respondents were asked to recall antibiotic use in the previous 12 months. The convenience sampling approach was applied in administration of the number of structured questionnaires to be given to the university and each LGA. Approximately 400 questionnaires were given to nominated undergraduate students or research assistants to administer to halls of residence. At the LGA, each health care facilitator was given 50 questionnaires per LGA to administer to the community members.

### 2.3. Data Analysis

Each health care facilitator at the LGA and nominated undergraduate students’ research assistant returned the questionnaires to the principal investigator. Data was accumulated and analysed with Microsoft excel and Stata version 12 software (College Station, TX, USA) to generate differential descriptive statistics including frequency tables that showed the frequency and percentage distribution of observations with categorical responses. Bar charts to illustrate responses were drawn using GraphPad Prism 5 for Windows (La Jolla, CA, USA). All of the data used in this study has been made available at www.osf.io.

### 2.4. Ethical Considerations

This study was conducted following the general principles of the Helsinki declaration [19]. This study did not apply any invasive approach on subjects, and informed consent was obtained from all participants. Institutional approval for the study was obtained from the Federal University Birnin Kebbi.

## 3. Results

### 3.1. Socio-Demographics of Respondents

A total of 1230 respondents (undergraduate students and community members), out of a total of 1450 questionnaires administered, partook in this cross sectional survey, representing 84.8% of the proportion of people that consented to participate and returned the questionnaires. The respondents comprised 42.6% males and 57.4% females in all. The male to female ratio was 1:1.34.

### 3.2. Socio-Demographic Characteristics among the University Students

Among the undergraduate students, female respondents were more represented than their male counterparts: 52% and 48% respectively (Table 1). The age groups of respondents were distributed as 20–25 years, 60%; 26–31 years, 33%; 14–19 years, 7%; 32–37 years and >37 years, <1%.

### 3.3. Socio-Demographic Characteristics of Community Members

Among the community members, female respondents were more represented than male: 60% and 40% respectively. The respondents were between the ages of 20–25 years (26%), 26–31 years (25%), and 32–37 years (19%). The occupations of the respondents were farmers (14%), housewives (22%), office job (30%), self-employed (19%), and unemployed (15%). Marital status of the respondents showed that 65% were married, 31% unmarried, and 4% divorced. With respect to their educational level, those with tertiary education comprised 57.5%; secondary education, 31%; primary education, 7%; and those who were illiterates, 5%, Table 2.

### 3.4. Frequency, Types and Place of Purchase of Commonly Used Antibiotics

**Figure 1:**
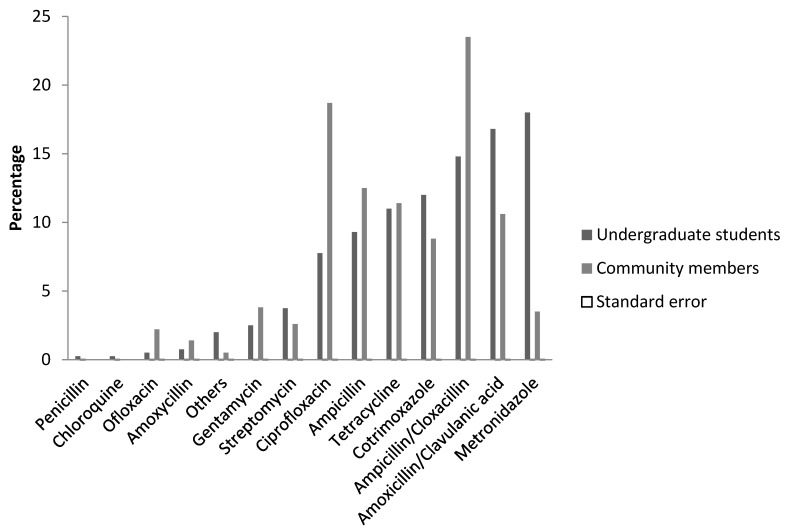
Commonly used antibiotics.

Frequency of antibiotic use among the undergraduate students and community members was reported as 43% and 26% weekly, respectively. Undergraduate students were more likely to use antibiotics as reported in the weekly usage of antibiotics (43%) compared to community members (26%). Prescription of antibiotics for use by a clinician, nurse, or pharmacist was 33.5%, 29%, and 25%, respectively, among undergraduate students and 57%, 20.4%, and 15.5% among the community members. Antibiotics purchase mostly took place at patent medicine stores (40%) among the undergraduate students, unlike the community members whose main source of purchase was a local chemist or pharmacy (48.4%). However, a substantial number of the undergraduate students compared to community members reported patronizing local drug hawkers (22.7% and 9.4% respectively). The question regarding compliance with the prescribed duration of use of the antibiotics indicated that 85% of undergraduate students and 84% of community members completed the course of antibiotic prescription. Similarly, the majority of the respondents (72.5% undergraduate students and 60.4% community members) suggested that antibiotic use was devoid of any side effects. In contrast, respondents reported different levels of satisfaction from the use of antibiotics. Undergraduate students mostly rated the antibiotic use as good (43%) while the community members mostly rated it as satisfactory (40%) in resolving illnesses (Table 3). The chi-square tests indicate significant association between frequency of antibiotic use, prescribing personnel, place of purchase, and efficacy of antibiotics in resolving illnesses with self-medication with antibiotics among the undergraduate students and community residents (*p*-value < 0.001). The distribution pattern of most commonly used antibiotics among undergraduate students was metronidazole (18%), amoxicillin/clavulanic acid (16.8%), ampicillin/cloxacillin (14.8%), cotrimoxazole (12%), and tetracycline (11%). Meanwhile the community members reported the highest use of ampicillin/cloxacillin (23.5%), ciprofloxacin (18.7%), ampicillin (12.5%), tetracycline (11.4%), and amoxicillin/clavulanic acid (10.6%), Figure 1.

### 3.5. Commonly Treated Illnesses

**Figure 2:**
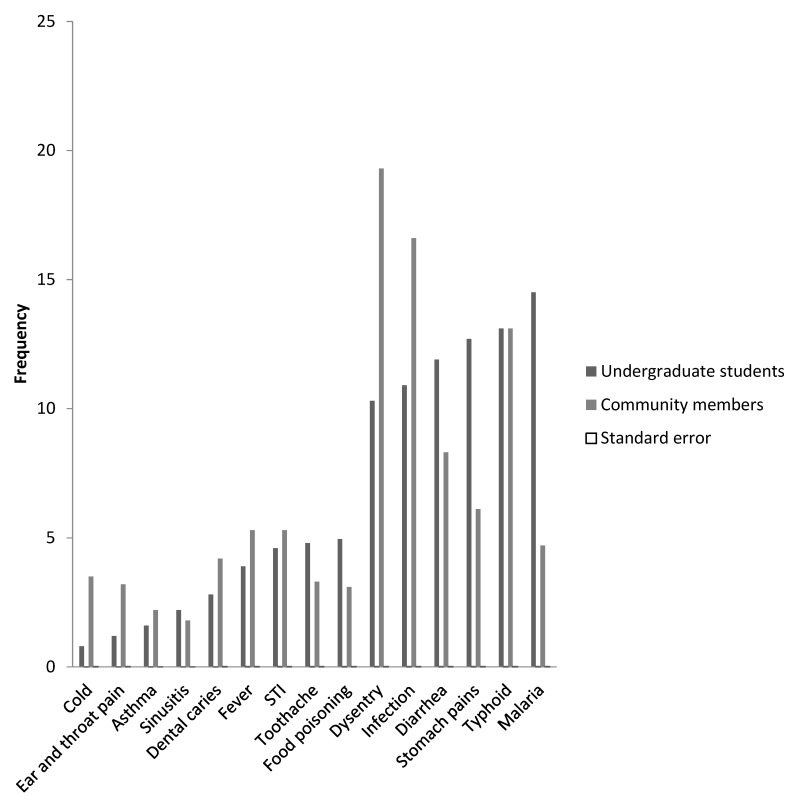
Comparison of commonly treated infections among community members and undergraduate students.

Responses from the undergraduate students suggest that the commonly self-diagnosed illnesses treated with antibiotics were malaria (14.5%), typhoid (13.1%), stomach pains (12.7%), diarrhea (11.9%), cold (0.8%), ear and throat pain (1.2%), asthma (1.6%), sinusitis (2.2%), dental caries (2.8%), and fever (3.9%). On the other hand, the community members were more likely to use antibiotics for illnesses such as dysentery (19%), infection (17%), typhoid (13%), sinusitis (1.8%), asthma (2.2%), food poisoning (3.1%), and ear and throat pain (3.2%), Figure 2.

### 3.6. Source of Antibiotic Choice

**Figure 3:**
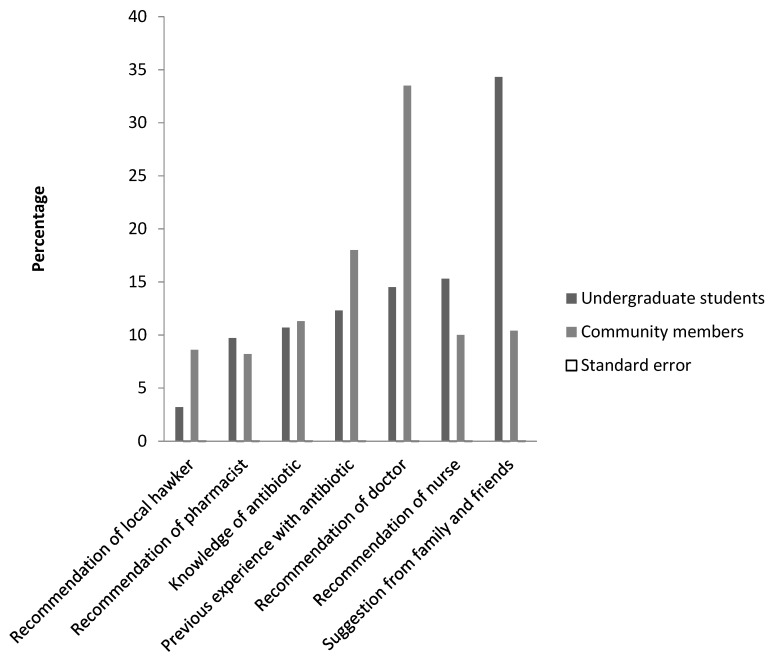
Sources of choice of antibiotics.

**Figure 4:**
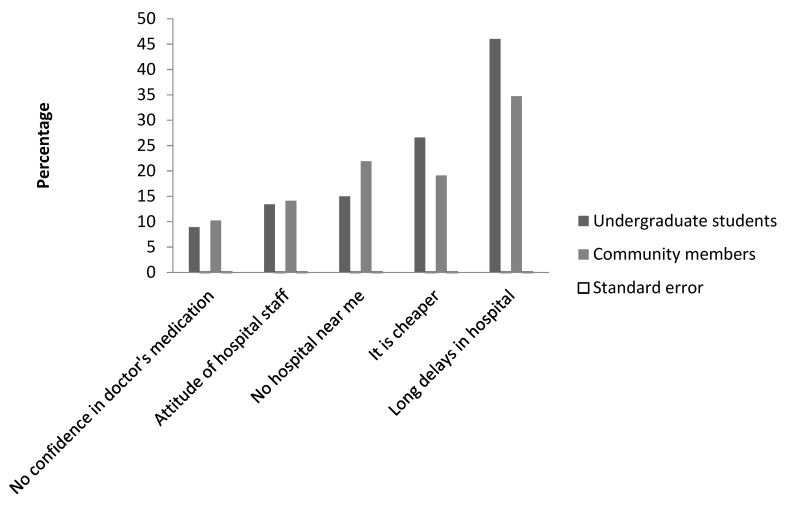
Reasons for self-medication with antibiotics.

Data collected from undergraduate students showed that suggestion from family and friends (34.3%) was the most common explanation for self-medication of self-diagnosed illnesses. In contrast, in the community, prescription by physicians (33.5%) was the highest response for choice of antibiotics, followed by previous experience with the antibiotic (18%), and knowledge of the antibiotic (11%) (Figure 3). Reasons reported for engaging in self-medication among undergraduate students and community members were due to long delays in the hospital (46% and 35%), it being cheaper not to go to hospital (26% and 19%), and the distance to the hospital (15% and 21%) (Figure 4).

### 3.7. Knowledge of Antibiotic Resistance among Undergraduate Students and Community Residents

Knowledge of ABR varied among the respondents. The undergraduate students, compared to the community residents, displayed more knowledge of the fact that antibiotics cannot cure all infections (53% and 42.4%), that antibiotics are not used for cold (57.5% and 55.3%), and that unnecessary use of antibiotics could make them ineffective (68.4% and 62.9%); the correct response to the questions was false. In contrast, community residents displayed better knowledge compared to undergraduate students when asked if antibiotics can be used for body pains (59.1% and 37.2%), and if antibiotics might be effective without completing the dosage (52.9% and 43.9%); the correct response was false. The undergraduate students, compared to the community residents, displayed less knowledge of the fact that self-medication could lead to development of ABR (32.6% and 42.2% respectively), but undergraduate students showed more knowledge that indiscriminate antibiotic use could cause ABR (51.3% and 41.8%) Table 4.

### 3.8. Attitude towards the Use of Antibiotics

Next, we reported the attitude of undergraduate students versus community residents with respect to antibiotic use. Our data suggests that the majority of the undergraduate students and community residents take antibiotics with water only (71% and 81.8% respectively). Other respondents in both groups admitted taking antibiotics with other liquids such as tea, juice, soft drinks, and others. The proportion of respondents who took antibiotics with other liquids was higher among the undergraduate students compared to the community residents: tea, 9.4% and 5%, respectively; juice, 4.4% and 3%; soft drinks, 10% and 5%; and other liquids, 5.2% and 5.2%.

Undergraduate students were more likely than the community residents to subscribe to using left over antibiotics from friends or family members without doctor’s prescription; most times (12.5% and 8.5%, respectively) and sometimes (57.4% and 40.3%). Similarly, the undergraduate students were more likely than the community residents to use antibiotics based on relative’s advice; always (25.4% and 18.3%, respectively) and sometimes (61.4% and 37.7%), summarized in Table 5.

## 4. Discussion

This study set out to investigate the patterns of self-medication, attitude towards antibiotic use, and knowledge of ABR among undergraduate students enrolled in a federal university and community members across the 21 LGAs in Northwest Nigeria. Here, we show that undergraduate students were more involved than community members in self-medication with antibiotics. In addition, it was observed that 43% of the undergraduates reported weekly usage of antibiotics for self-diagnosed illnesses, in contrast to 27% of community members that reported monthly usage. This trend is quite disturbing considering the frequency of antibiotics use and the huge potential for development of ABR by bacteria, and the preponderance of fake/substandard drugs in the market in Nigeria. Constant exposure to low doses of antibiotics may alter the gut flora and increase the risk of urinary tract infections, as reported among young women of child bearing age [20]. The high prevalence of self-medicated antibiotics by undergraduates in this study is consistent with findings amongst undergraduate students in Zaria, where 75.9% were engaged in self-medication with antibiotics [21]. A study in southwestern Nigeria in a rural population observed that 82.2% of the respondents practiced self-medication with antibiotics, in contrast to 52% reported amongst community respondents in this study [12]. In Zaria, the main reason for self-medication with antibiotics by undergraduates was assumed knowledge of the antibiotics by 35% of the respondents [21]. Amongst Northwest undergraduate students, the data here showed that the primary reason was the long delays in the clinic, as reported by 46% of the students. The pattern of self-medication with antibiotics amongst community members in Northwest Nigeria is much lower than that observed in the south and could be attributed to economic reasons. In a study carried out in Southwestern Nigeria, the cost of going to the hospital was given as a major reason for self-medication by 10.8% of the respondents, which is in contrast to data from Northwest which suggested long delays encountered in hospitals [22]. Health care in Northwest Nigeria is heavily subsidized by the state governments due to the high level of poverty, low income levels, and illiteracy compared to the Southern part of Nigeria. This might explain the difference in reasons provided for self-medication in these two regions of the country. In general, self-medication with antibiotics is a frequent event in Nigeria as with other developing countries. For example self-medication with antibiotics has been documented in Rajshahi, Bangladesh (26.69%) [23], and Karachi (47.6%) [24].

Furthermore, it was found that the most commonly used antibiotics amongst undergraduate students and community members were metronidazole and ampicillin/cloxacillin, respectively. In addition, we observed a high prevalence of ciprofloxacin self-medication amongst community members compared to undergraduate students. The report of metronidazole as the most commonly self-prescribed antibiotic amongst undergraduates is similar to reports in Bangladesh [23]. Similar studies in Mongolia, Arab Emirates, and Nigeria have indicated penicillins (ampicillin and amoxicillin) were commonly used for treatment of various illnesses [21,25,26]. Of note is a high prevalence of ciprofloxacin (fluoroquinolones) amongst members of the community. Indiscriminate use of fluoroquinolones has resulted in the spread of fluoroquinolones resistant enteric pathogens in Nigeria and other developing countries, making empiric treatment of illnesses such as typhoid and other bacterial infections with first line antibiotics more challenging [27,28]. Our cross sectional survey revealed that malaria, typhoid, stomach pains, diarrhea, and dysentery were the most common illnesses for which self-medicated antibiotics were used amongst undergraduate students and community members. Among social science university students in Northwest Ethiopia, it was observed that headache was the common symptom for indiscriminate use of antimicrobials [29].

Of note is the fact that only 15% of the undergraduate students received prescription antibiotics from physicians in contrast to 33.5% of community residents. This significant outcome of university students engaging more in self-medicated antibiotics is consistent with results obtained in Zaria and amongst Southwest university female students, where 1 in 4 women were found to self-medicate [21,30]. The long delays in hospital was the singular most important reason adduced to self-medication with antibiotics by undergraduate students and community residents. Health care systems in Nigeria and developing countries are sub optimal and patients usually experience long waiting times [31], with poor customer service from the health care workers. The long delays in hospital has also been cited as a reason for self-medicated antibiotics by undergraduate students in Zaria and Northwest Ethiopia [21,30]. Our data here also suggests that undergraduate students were more likely to engage in self-medication than community residents; this might be due to their level of education and confidence in the knowledge of the antibiotic being self-medicated. This observation is similar to findings among the Yoruba ethnic group in Southwest Nigeria, where education was indicated as a risk factor for self-medicated antibiotics; it was also descriptively associated with the marital status and level of education [32]. Our study also revealed that a major source of antibiotic prescription was from family and friends for university students, while there was a higher response rate for community residents seeking doctors’ prescription. This observation presents two contrasting events occurring within the state. Understanding the sources of antibiotics self-medication could assist public health practitioners to design community-based interventions to reduce the burden of antibiotic self-medication and improve measures for regulatory control of antibiotics sales. The apathy towards prescription antibiotics by university students attributed to long delays might also be due to inaccessibility of the university clinic to most of the undergraduate students. Most antibiotic purchases by the undergraduate students and community members were carried out in patent medicine stores that are not licensed to sell antibiotics or prescription medicines. This brings to light the indiscriminate sale of antibiotics and lack of tight regulatory control of antibiotic sales in Nigeria. Indiscriminate sales of antibiotics increase the chances of self-medicating antibiotics, an increase in ABR, and the preponderance of counterfeit antibiotics in the market.

In addition, there is a need for a multifaceted approach to tackle the issues identified as predisposing to self-medicated antibiotics in Nigeria. First, is the need for efficient implementation of laws already in place to ensure that patent medicine stores do not sell prescription only medicines. Second, improving the waiting times and efficiency of health facilities in Nigeria in order to encourage more hospital visits by sick people. Third, at the community level there needs to be appropriate public health education to inform the public on the dangers of self-medicated antibiotics since family and friends are likely sources of antibiotic choice [33].

The high burden of ABR remains a huge concern in developing countries and globally, as 700,000 lives are lost annually to ABR [15]. In Africa, there is widespread abuse of antibiotics, with over 80% of antibiotics used not prescribed; this pattern has led to a surge in ABR [34]. Self-medication with antibiotics is positively correlated to inappropriate antibiotic use and ultimately to development of ABR [35]. To curtail this trend the world health assembly in May 2015 made it its number one objective to increase awareness and understanding of ABR throughout the world [3]. To this end, we tested the knowledge of ABR in our setting using close ended questions. The data collected revealed mixed responses on knowledge of ABR between undergraduate students and community members. About 67% and 53% of undergraduate students and community members respectively indicated that they had heard of ABR. Undergraduate students displayed better knowledge in questions such as whether antibiotics can cure all infections, the use of antibiotics for cold, and that unnecessary use of antibiotics could make them ineffective, compared to community members. Conversely, community members displayed less knowledge of the fact that self-medication could lead to development of ABR, but undergraduate students showed more knowledge that indiscriminate antibiotic use could cause ABR. In both groups interviewed, we observed that the least knowledge was of the link between self-medication and ABR, when compared to correct responses from other questions. The data suggests that there is a gap in the understanding of the respondents that self-medication with antibiotics could result in development of ABR. The results from this study compared with a multi-country study commissioned by the WHO on 9772 adults with different educational levels drawn from 12 different countries with lower (Egypt, India, Indonesia, Nigeria, Sudan, Vietnam) or higher (Barbados, China, Mexico, Russian Federation, Serbia, South Africa) incomes [3].

To our knowledge this is the first study investigating the knowledge of ABR in Nigeria. This was a cross-sectional study that utilized a self-administered survey to estimate the prevalence of self-medicated antibiotic use, knowledge of ABR, and attitude towards antibiotic use. Hence, there is a likelihood of recall bias that cannot be ruled out. In addition, since the survey was self-administered, respondents may have skipped questions that they did not understand or given inaccurate information. Another limitation of this study was that, amongst undergraduates, more males were predisposed to filling in the questionnaires; this may affect the pattern of antibiotics used and illnesses commonly treated. The high illiteracy of this setting, with less than 50% being able to speak and write in the English language was also another challenge encountered during questionnaire administration. We also employed a random sampling technique based on convenience to obtain data from community members in all the 21 LGAs in Kebbi. Hence, it might be premature to conclude that our data could be applied to the whole of Nigeria.

## Figures and Tables

**Table 1:** Characteristics of the undergraduate students.

Characteristics	Frequency (N)	Percentage (%)	Characteristics	NDHS 2013 Men (%)	NDHS 2013 Women (%)
**Sex**			**Sex**		
Male	173	48	Male	NA	NA
Female	185	52	Female	NA	NA
Total	358	100	Total		
**Age group**			**Age group**		
14–19	23	7	15–19	20.9	20.1
20–25	204	60	20–24	16.7	17.3
26–31	111	33	25–29	15.9	18.3
32–37	1	0.3	30–34	13.9	14.0
Others	1	0.3	35–39	12.5	12.1
Total	340	100			
**Academic Year**			**Level of education**		
1	122	35	No education	21.2	37.8
2	138	40	Primary	16.7	17.3
3	88	25	Secondary	47.7	35.8
Total	348	100	More than secondary	14.3	9.1

NA—Not Applicable; NDHS—Nigeria Demographic and Health Survey.

**Table 2:** Characteristics of community members.

Characteristics	Frequency (N)	Percentage (%)	Characteristics	NDHS 2013 Men (%)	NDHS 2013 Women (%)
**Sex**			**Sex**		
Male	351	40	Male	NA	NA
Female	521	60	Female	NA	NA
Total	872	100	Total		
**Age group**			**Age group**		
14–19	82	9.5	15–19	20.9	20.1
20–25	228	26	20–24	16.7	17.3
26–31	216	25	25–29	15.9	18.3
32–37	161	19	30–34	13.9	14.0
38–43	118	13.6	35–39	12.5	12.1
44–49	50	5.8	40–44	10.2	9.3
>49	11	1.3	45–49	9.9	8.8
Total	866	100			
**Occupation**			**Occupation**		
Farmer	111	14	Agriculture	33.7	15.6
Housewife	174	22	Unskilled manual	4.9	0.5
Office job	241	30.5	Professional/technical	11.3	7.4
Self-employed	150	19	Skilled manual	23.0	14.3
Unemployed	115	14.5			
Total	791	100			
**Marital status**			**Marital status**		
Divorced	34	41	Divorced/separated	1.2	2.1
Married	537	65	Married	49.1	69.4
Single	255	31	Never married	48.3	23.9
Total	826	100			
**Educational status**			**Level of education**		
None	40	4.7	No education	21.2	37.8
Primary	59	7	Primary	16.7	17.3
Secondary	260	30.8	Secondary	47.7	35.8
Tertiary	484	57.5	More than secondary	14.3	9.1
Total	843	100			

NA—Not Applicable; NDHS—Nigeria Demographic and Health Survey.

**Table 3:** Characteristics of undergraduate students and community members indulging in self-medicated antibiotics.

Characteristics	Undergraduates	Community Members	Chi-Square
	N (%)	N (%)	
**Frequency of antibiotics use**			
Weekly	150 (43.2)	231 (26.3)	
Monthly	60 (17.3)	237 (27)	
Once in two months	65 (18.2)	147 (16.7)	53.3 ***
Every 3 months	53 (15.3)	128 (14.6)	
Others	19 (6)	137 (15.6)	
Total	347 (100)	880 (100)	
**Who prescribed it?**			
Doctor	65 (33.5)	411 (57)	
Nurse	56 (28.9)	147 (20.4)	
Pharmacist	49 (25.2)	112 (15.5)	34.6 ***
Others	24 (12.4)	51 (7.1)	
Total	194 (100)	721 (100)	
**Place of purchase**			
Local chemist	100 (33.3)	350 (48.4)	
Patent medicine store	119 (39.7)	297 (41.1)	
Local drug hawkers	68 (22.7)	68 (9.4)	49.9 ***
Others	13 (4.3)	8 (1.1)	
Total	300 (100)	723 (100)	
**Did you comply with the duration of use of the antibiotics?**			
Yes	288 (85.2)	705 (83.5)	
No	50 (14.8)	139 (16.5)	0.50
Total	338 (100)	844 (100)	
**Were there any side effects?**			
Yes	92 (27.5)	322 (39.6)	
No	243 (72.5)	492 (60.4)	15.0 ***
Total	335 (100)	814 (100)	
**Was the antibiotic effective in resolving the illness?**			
Excellent	46 (13.7)	172 (21.1)	
Satisfactory	85 (25.3)	327 (40.2)	
Good	146 (43.4)	274 (33.7)	70.8 ***
No results	59 (17.6)	41 (5)	
Total	336 (100)	814 (100)	

*** *p*-value < 0.001.

**Table 4:** Frequency and percentage distribution of knowledge of antibiotics resistance.

Characteristics	Undergraduates N (%)	Community Residents N (%)	Chi-Square
**Antibiotics can cure all infections**			
True	170 (47)	509 (57.6)	11.8 **
*False*	192 (53)	374 (42.4)	
Total	362 (100)	883 (100)	
**Antibiotics can be used for cold**			
True	188 (52.4)	440 (50)	0.69
*False*	171 (47.6)	444 (50)	
Total	359 (100)	884 (100)	
**When I have cold, I should always take antibiotics**			
True	153 (42.5)	392 (44.7)	0.50
*False*	207 (57.5)	485 (55.3)	
Total	360 (100)	877 (100)	
**Antibiotics can be used for body pains**			
True	226 (62.8)	357 (40.9)	49.0 ***
*False*	134 (37.2)	516 (59.1)	
Total	360 (100)	873 (100)	
**Antibiotics might be effective even if I don’t complete the dosage**			
True	202 (56.1)	412 (47.1)	8.3 **
*False*	158 (43.9)	463 (52.9)	
Total	360 (100)	875 (100)	
**Unnecessary use of antibiotics makes them ineffective**			
*True*	247 (68.4)	542 (62.9)	3.4
False	114 (31.6)	320 (37.1)	
Total	361 (100)	862 (100)	
**Have you heard of resistance to antibiotics**			
Yes	239 (67)	453 (53.4)	18.8 ***
No	118 (33)	395 (46.6)	
Total	357 (100)	848 (100)	
**Resistance to antibiotics can be caused by**			
*Self-medication*	85 (32.6)	310 (42.2)	8.5 *
*Not completing the dosage*	42 (16.1)	118 (16)	
*Using antibiotics repeatedly*	134 (51.3)	307 (41.8)	
Total	261 (100)	735 (100)	
**Level of awareness of antibiotics resistance**			
Low	31 (12.1)	65 (10)	11.6 **
Moderate	156 (60.7)	478 (72)	
High	70 (27.2)	121 (18)	
Total	257 (100)	664 (100)	

* *p*-value < 0.05, ** *p*-value < 0.01, *** *p*-value < 0.001. Italicized responses are correct answers to questions asked.

**Table 5:** Attitude towards antibiotics among community residents and undergraduate students.

Characteristics	Undergraduates N (%)	Community Residents N (%)	Chi-Square
**With what do you take your antibiotics?**			
Water	242 (71)	718 (81.8)	22.1 ***
Tea	32 (9.4)	44 (5)	
Juice	15 (4.4)	26 (3)	
Soft drinks	34 (10)	44 (5)	
Others	18 (5.2)	46 (5.2)	
Total	341 (100)	878 (100)	
**Do you use left-over antibiotics from friends or family without doctor’s consultation?**			
Always	42 (12.5)	73 (8.5)	43.7 ***
Sometimes	193 (57.4)	347 (40.3)	
Never	101 (30.1)	441 (51.2)	
Total	336 (100)	861 (100)	
**Do you use antibiotics based on relative’s advice?**			
Always	89 (25.4)	160 (18.3)	105.4 ***
Sometimes	215 (61.4)	329 (37.7)	
Never	46 (13.2)	384 (44)	
Total	350 (100)	873 (100)	
**Do you use another family member’s antibiotics?**			
Always	60 (17.2)	121 (13.8)	73.0 ***
Sometimes	197 (56.5)	294 (33.5)	
Never	92 (26.4)	462 (52.7)	
Total	349 (100)	877 (100)	

*** *p*-value < 0.001.
